# Modeled Structure of the Cell Envelope Proteinase of *Lactococcus lactis*

**DOI:** 10.3389/fbioe.2020.613986

**Published:** 2020-12-22

**Authors:** Egon Bech Hansen, Paolo Marcatili

**Affiliations:** ^1^National Food Institute, Technical University of Denmark, Kongens Lyngby, Demark; ^2^Department of Health Technology, Technical University of Denmark, Kongens Lyngby, Demark

**Keywords:** lactic acid bacteria (LAB), cell envelope associated peptidases, protein structure prediction, proteolytic system, casein micelle, subtilisin, S8 proteinase, substrate specificity

## Abstract

The cell envelope proteinase (CEP) of *Lactococcus lactis* is a large extracellular protease covalently linked to the peptidoglycan of the cell wall. Strains of *L. lactis* are typically auxotrophic for several amino acids and in order to grow to high cell densities in milk they need an extracellular protease. The structure of the entire CEP enzyme is difficult to determine experimentally due to the large size and due to the attachment to the cell surface. We here describe the use of a combination of structure prediction tools to create a structural model for the entire CEP enzyme of *Lactococcus lactis*. The model has implications for how the bacterium interacts with casein micelles during growth in milk, and it has implications regarding the energetics of the proteolytic system. Our model for the CEP indicates that the catalytic triad is activated through a structural change caused by interaction with the substrate. The CEP of *L. lactis* might become a useful model for the mode of action for enzymes belonging to the large class of S8 proteinases with a PA (protease associated) domain and a downstream fibronectin like domain.

## Introduction

Lactic acid bacteria (LAB) are commonly found in ecological niches rich in nutrients. The adaption to nutrient rich environments has resulted in extensive genome reduction and fastidious growth requirements including the demand for multiple amino acids (Jensen and Hammer, 1993; Makarova et al., 2006; Gänzle, 2015). In nutrient rich environments LAB compete against other microorganisms by fast growth and rapid accumulation of lactic acid from carbohydrate fermentation. The demand for essential amino acids will typically lead to a depletion of low molecular weight peptides and free amino acids. Growth beyond this point requires protein hydrolysis (Juillard et al., 1995b; Vermeulen et al., 2005). Proteolytic phenotypes are widespread among LAB (Kliche et al., 2017) whereas also the strategy of relying on symbiosis with proteolytic strains seems to be competitive (Sodini et al., 2000; Bachmann et al., 2012). The proteolytic system of LAB consist of an extracellular proteinase, various transporters allowing peptides and amino acids to be imported into the cell, and a range of intracellular peptidases completing the hydrolysis of peptides into amino acids (reviewed by Savijoki et al., 2006). The extracellular proteinase is typically a large serine proteinase attached to the cell envelope with a proteinase domain homologous to the *Bacillus* protease subtilisin (Siezen, 1999; Siezen and Leunissen, 2010; Broadbent and Steele, 2013).

The proteinases of LAB used in the dairy industry have been studied for two main reasons: speed of acidification and flavor formation. Particularly the *Lactococcus lactis* cell envelope proteinases (CEP) have been extensively studied. The *L. lactis* CEP is a large multi domain enzyme covalently attached to the peptidoglycan layer at the outside of the cell wall (Siezen, 1999). The CEPs from different strains of *L. lactis* differ very little in sequence with identities larger than 98%. However, the enzymes differ regarding flavor development and substrate specificity (Visser et al., 1986; Exterkate, 1990; Exterkate et al., 1993; Broadbent et al., 1998; Exterkate and Alting, 1999; Børsting et al., 2015).

It was recently demonstrated that proteolysis is the bottleneck for acidification of camel milk with commercially available dairy starter cultures (Berhe et al., 2018). LAB strains able to acidify camel milk have been isolated (Abdelgadir et al., 2008; Drici, 2008; Drici et al., 2010; Gabed et al., 2015; Fugl et al., 2017) and several of these have been genome sequenced (Drici, 2008; Gabed et al., 2015; Bragason et al., 2020).

Comparison of CEP from strains of *Lactococcus lactis* with different properties toward caseins from camel and cow milk is likely to reveal fundamental properties of this important enzyme. Knowledge of the structure of the protein is useful for the fundamental understanding of an enzyme. In this paper we describe the development of a structural model of the PrtP enzyme starting from the amino acid sequence of PrtP from *Lactococcus lactis* MS22337, a strain isolated from spontaneously acidified camel milk.

The architecture of the *L. lactis* CEP shares features with a large number of serine proteases. The subtilisins annotated in the Pfam database^1^ are characterized by the shared domain S8 (PF00082). Domain S8 is found in 43564 sequences and a large class of those contain an insertion of a protease associated domain, PA(PF02225) within the S8 domain. Among those, there is often a fibronectin-like domain just downstream for the S8 domain. In prokaryotic proteases the fibronectin-like domain has been termed Fn3_5(PF06280). Fn3_5 is found in 1515 sequences and always together with S8. In plant proteases, the fibronectin-like domain is termed Fn3_6(PF17766) and this domain is found in 5862 sequences. The possible interaction between PA, S8, and Fn in the CEP of *L. lactis* might reveal a common principle used by this large class of proteases. The objective of this study is to use current state of the art structure prediction algorithms to establish a structural model of the CEP of *L. lactis* and to examine if the predicted model have implications for the function of the enzyme.

## Materials and Methods

### CEP Sequences

The *prtP* genes of *Lactococcus lactis* strains Wg2 and SK11 were the first CEP genes to be cloned and sequenced (Kok et al., 1988; Vos et al., 1989a). The amino acid sequences of these two enzymes are available in Uniprot under the accession numbers P16271 and P15292 respectively. We did however during this work realize that the sequence at Uniprot accession P15292 deviates from the sequence given in the original papers and several subsequent papers on SK11 PrtP. We therefore used the PrtP sequence translated from the DNA sequence for the complete proteinase plasmid pSK11P with accession DQ149245 (Siezen et al., 2005). The two sequences for the same protein differ in 23 positions. We also compared the 2005 PrtP sequence to the original SK11 PrtP sequence from 1989 (Vos et al., 1989a) and found deviation at 3 positions, I109T, S592F, and D899E, of which the first is located in the pro-peptide. In this paper we also use the sequences of the CEP enzymes from two *Lactococcus lactis* subsp. *lactis* strains MS22333 and MS22337 isolated from spontaneously acidified camel milk in Ethiopia (Fugl et al., 2017), these two sequences have been deposited at the NCBI GenBank under the accession numbers WWDI00000000 and WWDK00000000 respectively. An alignment of the four PrtP amino acid sequences is given in Supplementary Figure S1. The two strains isolated from camel milk differ from each other in 39 positions and with Wg2 in 50 and 54 positions respectively. They show the largest difference to the SK11 PrtP where differences are found for 68 and 73 positions within the first 1800 amino acids. In the last domain, W, composed of a variable number of a 60 aa repeat unit we see differences of up to seven amino acids between repeat units from different strains and up to two differences in amino acid sequence between units from the same strain.

### Protein Structure Modeling

Protein structures were modeled using the four different methods given in Table 1. Restrictions on sequence length apply for I-TASSER and RaptorX forcing the modeling of large proteins to be conducted in segments. Swiss-Model has a feature allowing a template to be provided for the modeling. This feature was used to impose the repeated use of the same template on a longer segment of the protein sequence. The PyMOL software (version 2.3.3)^2^ was used for visualization and editing of protein structure PDB files. Comparison of protein structures and calculation of TM-scores and RMSD values was done using the TM-score and TM-align programs at University of Michigan^3^ (Zhang and Skolnick, 2004). Individual domains were assembled into larger models using the DEMO program (Zhou et al., 2019).

**TABLE 1:** Algorithms used for structure prediction.

Algorithm	Server address	Reference
**Phyre2**	http://www.sbg.bio.ic.ac.uk/~phyre2	Kelley et al., 2015
**Swiss-Model**	https://swissmodel.expasy.org/	Waterhouse et al., 2018
**I-TASSER**	https://zhanglab.ccmb.med.umich.edu/I-TASSER/	Yang et al., 2015
**RaptorX**	http://raptorx.uchicago.edu/	Wang et al., 2017; Xu, 2019

## Results – Modeling the Structure of CEP

### One Step Modeling of CEP

The *L. lactis* PrtP enzyme starts from the N-terminal end with a signal peptide assuring the translocation of the protein across the membrane via the seg pathway (Cranford-Smith and Huber, 2018). Following the signal peptide comes a long propeptide which is autocatalytically cleaved by the PrtP enzyme to make an aspartic acid at position 188 to become the N-terminus of the mature enzyme (Kok et al., 1988; de Vos et al., 1989; Haandrikman et al., 1989; Vos et al., 1989a). We find a two amino acid deletion in the pro-peptide of PrtP enzymes in all *L. lactis* isolates from Ethiopian camel milk. In our sequence the N-terminal D residue thus has the residue number 186 rather than 188.

Two of the three modeling servers accept long amino acid sequences and as a first approach we attempted to model the entire PrtP molecule. The sequence of the mature PrtP protease from strain MS22337 was submitted to the servers of Phyre2 and Swiss Model. The sequence submitted was starting with the sequence DAK predicted to be the first amino acids in the mature PrtP protein after autocatalytic cleavage of the pro-peptide. The C-terminus of the sequence was the PKT predicted to be covalently linked to the peptidoglycan of the cell wall. The entire length of the protein sequence is 1725 amino acids.

Protein structures identified as best templates by the modeling servers are listed in Table 2.

**TABLE 2:** Protein structures identified as best templates for modeling the structure of the sequence of MS22337 PrtP.

Phyre2	Swiss Model	iTASSER	Protein	Reference
**Protease domain**				
5xyr/5xxz	5xyr/5xya		*Streptococcus pyogenes* ScpC virulence protease	Jobichen et al., 2018
1xf1	1xf1	1xf1	*Streptococcus agalactiae* C5a peptidase	Brown et al., 2005
	3eif	3eif	*Streptococcus pyogenes* C5a peptidase	Kagawa et al., 2009
		1r6v	Fervidolysin, *Fervidobacterium pennivorans*	Kim et al., 2004
		3vta	Cucumisin, *Cucumis melo*	Murayama et al., 2012
**A + B domain**				
4p99	4p99	4p99	MpAFP, *Marinomonas primoryensis*	Vance et al., 2014
2yn5			SiiE, *Salmonella enterica*	Griessl et al., 2013
		4rm6	HxuA, *Haemophilus influenzae*	Zambolin et al., 2016
		5ftx	SbsC, *Geobacillus stearothermophilus*	https://www.rcsb.org/structure/5ftx
		4om9	Pet passenger domain, *Escherichia coli*	Domingo Meza-Aguilar et al., 2014

*The actual structures can be downloaded from the Protein Data Bank at https://www.ebi.ac.uk/pdbe/.*

Although Swiss model accepts the entire sequence as input, the output is a model for only the first half of the sequence containing 912 amino acids ending at position 1100. Phyre2 also accepts the entire sequence as input and proposes the model shown in Supplementary Figure S2. In the Phyre2 model the first 1308 amino acids have been assigned a structure quite similar to the Swiss model, where the remaining 415 amino acids, for which no template has been identified, are modeled as random coils.

Phyre2 and Swiss model both use two streptococcal serine proteinases as templates for the structure models of the first half of the molecule. Both programs detect homology to a protein from *Marinomonas primoryensis* with the structure given by 4p99. The homology to this protein is found in the region from 996 to 1396 in the PrtP sequence. Phyre2 also detects a homology to 2yn5 in the region 1212–1496.

From the analysis of the templates identified by these tools and of the resulting models, we can see that the C-terminal region of the protein has not been modeled with sufficient accuracy, and additional remote homology templates and modeling constraints are needed to improve the modeling confidence.

### Identification of Domains of CEP by Evolutionary Information

**FIGURE 1:**
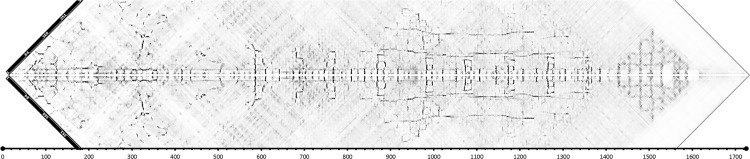
Contact map for *L. lactis* MS22337 PrtP generated by RaptorX by superimposing 799 aa long segments of the PrtP sequence starting from the first aa of the mature protein (D186). As the map covers only the region around the diagonal, the composite map has been tilted by 45°. The four segments are 1–799, 301–1099, 501–1299, and 926–1725 (in this figure, positions are relative to D186).

In recent years, evolutionary constraints (Marks et al., 2012) have revolutionized the field of protein modeling, by introducing the ability to identify long- and short-term interactions between residues solely on the base of residue conservation and co-variation as observed in multiple sequence alignments. RaptorX exploits such methods to predict contacts and use additional information for the identification of remote homologs. In order to use modeling servers with restriction on the length we need to split the PrtP sequence into sections. The split can be done according to assumed domains or just random in overlapping segments. The RaptorX server allows contact maps to be generated for segments up to a length of 800 amino acids. A number of overlapping maps were generated and the contacts predicted were independent on how the protein sequence was segmented. By superimposing the maps for overlapping segments it is possible to generate a “full length diagonal” contact map for the amino acids separated by less than about 200 amino acids as shown in Figure 1.

The contact map allows us to identify regions of the protein with distinct structural features. The first 520 amino acid residue region is predicted to have numerous short range and long-range contacts. This region has previously been recognized as the protease domain with homology to subtilisin (Siezen, 1999). After the protease domain comes a long region where antiparallel β-sheets seems to be a characteristic feature. Antiparallel β-sheets are characteristic for fibronectin like domains. Fibronectin domains are quite common among the subtilisins and for the bacterial subtilisins the domain has been termed Fn3.5 (see footnote 1). In the structure of the *Streptococcus pyogenes* C5a protease three fibronectin like domains were identified downstream for the protease domain (Kagawa et al., 2009; Kagawa and Cooney, 2013). In the region between aa 500 and 1400, Figure 1 shows a pattern which seems to be repeated nine times with intervals of about 100 aa. Each of these 9 patterns might represent individual fibronectin like domains. The three first units seem to be different from the last six and particularly the second unit seems to be involved in short-range and long-range contacts. The last six units appear to have long-range interactions between parallel β-sheets in addition to short-range antiparallel interactions. The long-range interactions are between parallel β-sheets separated by about 100 or 200 aa. These interactions could suggest that the structure is able to adopt alternative configurations with parallel β-sheets organized into β-barrels.

After the region with the fibronectin like domains, the contact map of Figure 1 shows a region from 1400 to 1620 (1570–1800 relative to start of the reading frame) with patterns indicative of α helixes. The pattern is consistent with a bundle of α helixes shoving parallel and antiparallel interactions. This region coincides with the domain termed H for helix by Siezen (1999). The region following the H domain has no predicted interactions. This domain was termed W by Siezen, based on a hypothetical role as a cell wall spanning domain (Siezen, 1999).

### The Proteinase Domain

Phyre2 and Swiss model gave structure predictions of the MS22337 PrtP enzyme. In addition we also used iTASSER to obtain models for the proteinase domain. The minimal domain was modeled by presenting a 511 aa long sequence starting from D 186 in the MS22337 PrtP sequence. The output from iTASSER contains several possible structures and two models, iTAS_1 and iTAS_2, are used for further analysis. In addition to the minimal protease domain we also used iTASSER to model an extended version of the proteinase domain by presenting a sequence starting with the amino acid following the predicted cleavage site of the signal peptidase. The 1077 aa long sequence presented to iTASSER was starting at K32. This sequence covers the proteinase domain preceded by the pro-peptide and followed by the A domain. Two models, iTAS_3 and iTAS_4 are retained for further analysis.

**FIGURE 2:**
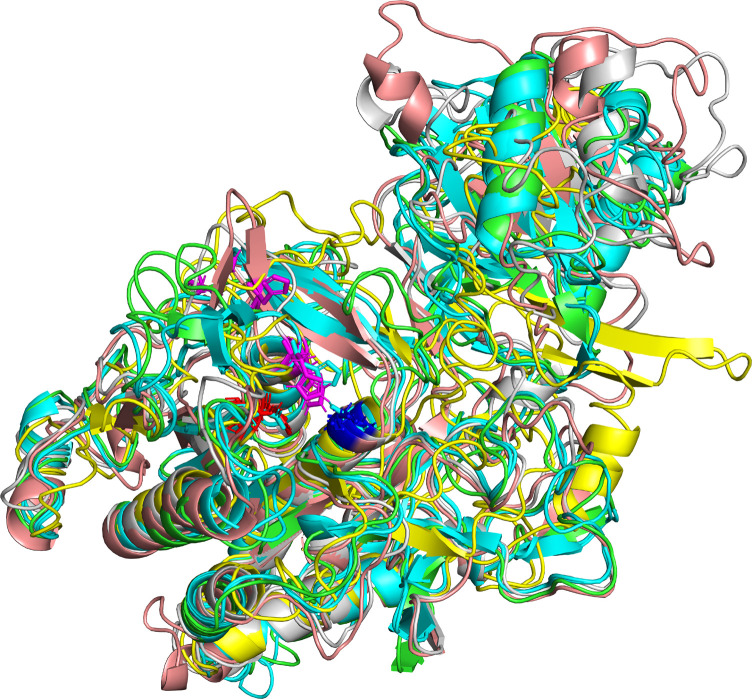
Predicted structures of the MS22337 PrtP proteinase for the domain spanning residue nr 186–698. The superimposed structures are shown for iTAS_1 in green, iTAS_3 in cyan, iTAS_4 in yellow, Phyre2 in salmon, and Swiss in gray. The protein backbones are shown as cartoons with the catalytic triad shown as stick models with D in red, H in magenta, and S in blue.

The four iTASSER models and the two models predicted by Phyre2 and Swiss model are compared in Table 3. When comparing only the structure of the “proteinase core” (residue 186-698) TM values are in the range from 0.55 to 0.95 indicating that that the predicted structures are in general consistent and belong to the same fold. The structure iTAS_4 is the structure differing the most from any of the other structures. iTAS_1, iTAS_2, and iTAS_3 are rather similar to each other and the Phyre2 and the Swiss models are also rather similar to each other. iTAS_2 seems to be the structure differing the least from any of the other structures within the “core proteinase region.” The five other structures were each aligned to the iTAS_2 structure and all structures were then superimposed using these five alignments. The modeled structures have been deposited at modelarchive.org^4, ^^5, ^^6, ^^7, ^^8, ^^9^. Figure 2 show the aligned structures for the “core protease region.”

**TABLE 3:** Comparison of protein structure models of *Lactococcus lactis* MS22337 PrtP.

		Model:					
“Core” Model:		iTAS_1	iTAS_2	iTAS_3	iTAS_4	Phyre2	Swiss
iTAS_1_I	Shared residues		513	513	513	513	511
	RMSD Å		3.666	4.393	14.084	7.568	7.251
	TM-score		0.916	0.439	0.303	0.250	0.440
iTAS_2_K	Shared residues	513		513	513	513	511
	RMSD Å	3.666		2.864	13.298	6.966	6.262
	TM-score	0.916		0.459	0.323	0.256	0.464
iTAS_3_M	Shared residues	513	513		1077	923	913
	RMSD Å	4.393	2.864		40.628	8.599	7.455
	TM-score	0.892	0.950		0.370	0.431	0.792
iTAS_4_O	Shared residues	513	513	513		923	913
	RMSD Å	14.084	13.298	13.374		21.854	22.044
	TM-score	0.559	0.608	0.606		0.244	0.395
Phyre2_P	Shared residues	513	513	513	513		913
	RMSD Å	7.568	6.966	6.191	14.516		6.859
	TM-score	0.739	0.773	0.791	0.573		0.812
Swiss_S	Shared residues	511	511	511	511	511	
	RMSD Å	7.251	6.262	6.332	14.325	5.879	
	TM-score	0.731	0.782	0.777	0.574	0.847	

*Models were compared using the TM-score program at the server of Zhanglab at University of Michigan (http://zhanglab.ccmb.med.umich.edu/TM-score). The values for each pairwise comparison are: the number of common residues; root-mean-square deviation (RMSD) of α-carbons; TM-score. The TM-score is a measure of structure similarity ranging from 0 to 1 (Zhang and Skolnick, 2004). The values above the diagonal (upper right) give the value for comparison of the “full length” models and the values below the diagonal (lower left) gives the value for comparing only the “proteinase core structure” (residue nr 186–698).*

#### The Catalytic Site

**FIGURE 3:**
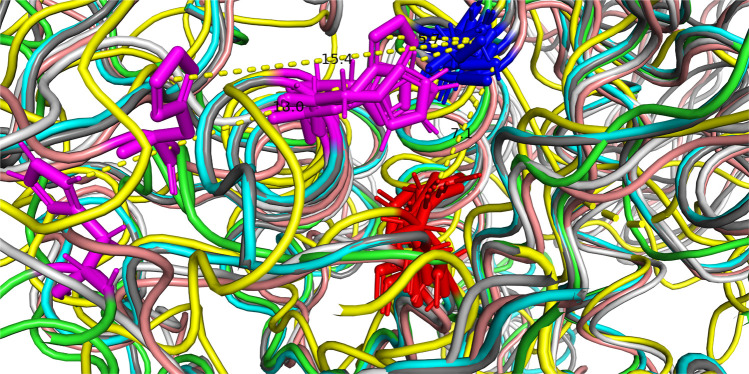
The catalytic triad of MS22337 PrtP in the six structural models predicted based on the amino acid sequence. Aspartic acid, D215, is shown in red; Histidine, H279, is shown in magenta; and Serine, S618, is shown in blue. The protein backbone of the α carbons are shown as tubes with different color: iTAS_1 in green, iTAS_2 in light blue, iTAS_3 in dark gray, iTAS_4 in yellow, Phyre2 in amber, and Swiss in light gray. All six structures have the aspartic acid (215) at the same position and they have the serine (618) at coinciding positions in a distance of 7 Å from the aspartic acid. The histidine (279) of four models (iTAS_2, iTAS_3, iTAS_4, and Swiss) are located a the same position in a distance of 6 Å from the two other amino acids of the triad, whereas the histidines of iTAS_1 and Phyre2 are located far from the active site with distances of 18 and 15 Å respectively to the S618.

**FIGURE 4:**
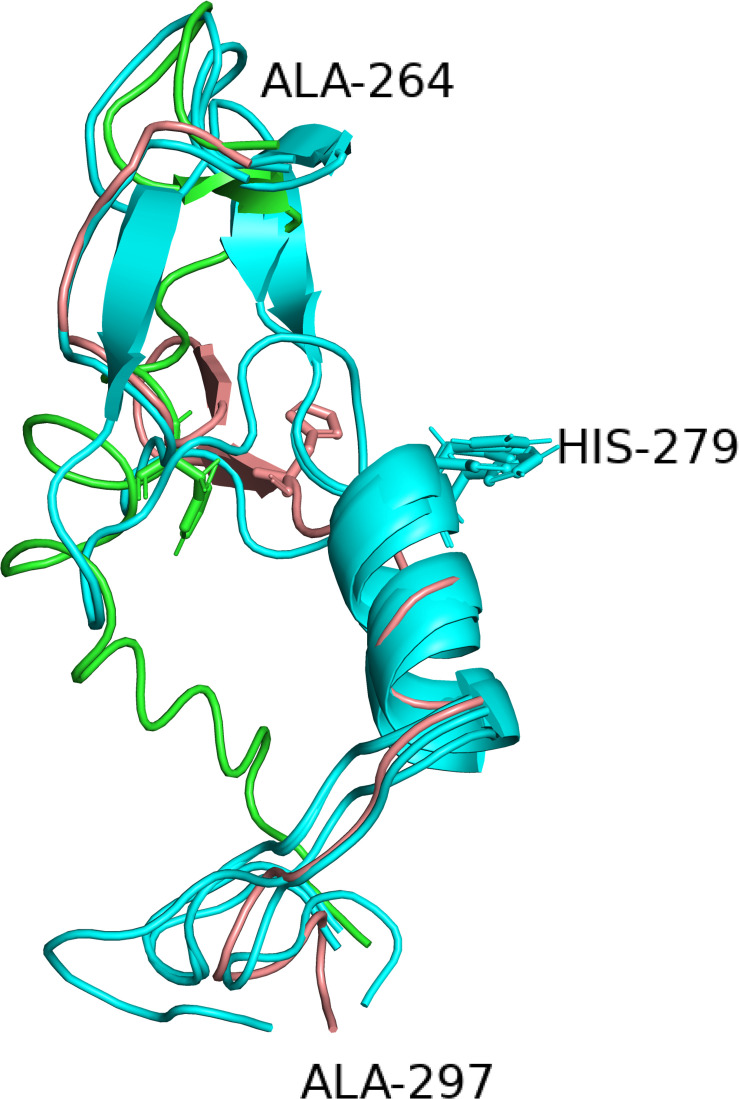
The alternative structures for the segment containing the histidine (279) of the catalytic triad. Four of the six structures have identical structures and are all shown in cyan whereas the two deviating structures iTAS_1 and Phyre2 are shown in green and salmon respectively. His 279s are shown as stick models in all structures.

An interesting aspect of the structure is the position of the amino acid residues of the catalytic site. The subtilisins (Pfam and Merops S8) are serine proteases with a catalytic triad consisting of three amino acids, aspartic acid, histidine, and serine. In the sequence of PrtP of MS22337 these amino acids are at the positions D215, H279, and S618. The relative positioning of these three amino acids in the six structures is given in Figure 3. It is remarkable that the aspartic acid and the serine residues, separated by 402 residues in the sequence, in all six models coincide at positions separated by seven Å. In four models; iTAS_2, iTAS_3, iTAS_4, and Swiss; the active site histidine is located at coinciding positions close to the two other amino acids of the catalytic triad. However, in the iTAS_1 and Phyre2 models, the histidine is located far from the active site. In these two models the histidine is displaced by 10–13 Å from the position presumably defining the position of the histidine in an active triad. It is tempting to speculate that the differences between the six models might reflect dynamics in the molecule and not only inaccuracy in the structure prediction. If this is the case, the different positions of the histidine of the catalytic triad might illustrate the possible mode of action of this enzyme. The enzyme could in the “ground state” be inactive due to a large distance between the histidine and the D and S residues. Recognition of a suitable substrate might lead to a change in structure moving the histidine to the active position in the active site. Figure 4 show the alternative structures for the region carrying the histidine 279.

#### Interaction With Substrate

**FIGURE 5:**
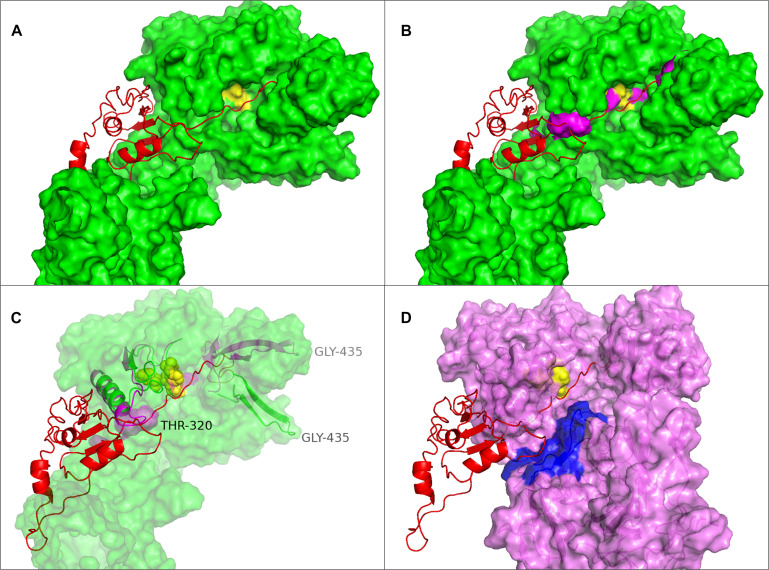
Substrate position in the catalytic cleft of PrtP. **(A)** The iTAS4 model. The pro-peptide (shown as cartoon in red) is positioned in the catalytic cleft on iTAS_4 (shown as green surface. The catalytic triad is shown in yellow. **(B)** The obstructions in path of substrate posed by the iTAS_3 model is shown as magenta surfaces in the same model as **(A)**. **(C)** The underlying change in structure allowing the substrate access to the catalytic cleft. Residue 320 is obstructing to one side and residue 429 is obstructing at the other site. The segments from 300–340 and 422–445 of iTAS_3 and iTAS_4 are shown as cartoons in magenta and green respectively inside the same surface as **(B)** (now transparent). **(D)** Possible substrate interaction in the area of fibronectin like structures. The surface of the iTAS_3 structure is shown in magenta. The position of substrate is shown in red and the catalytic triad is shown in yellow. The same obstacles for the substrate as seen in **(C)** is also obvious in this figure. The area in blue is a region within the fibronectin like domains spanning residue 930–950. This area might interact with larger substrates like entire caseins or long peptides.

By including the pro-peptide in the iTASSER modeling the two structures iTAS_3 and iTAS_4 were obtained as the two top ranking structures. The iTAS_3 model carries the pro-peptide as a long appendage whereas the pro-peptide in iTAS_4 is molded into the structure of the proteinase domain. The path of the pro-domain is passing the D and S of the catalytic triad in a distance of 4–8 Å from the amino acids of the catalytic triad. The path of the pro-peptide in this model seems to follow the catalytic cleft and thus defines the orientation of substrate bound to the protease. The pro-peptide is the very first substrate for the newly synthesized enzyme molecule and must therefore fit into the active site of the protease. In the iTAS_4 model the amino acid of the pro-peptide closest to the catalytic triad is residue number 161 whereas the activation is supposed to be a cleavage between residue 185 and 186. Figure 5A shows the position of the pro-peptide on the surface of the PrtP model iTAS_4. On the iTAS_4 model the pro-peptide (the substrate) follows a free trajectory along the length of more than 50 amino acids. On the iTAS_3 model (Figure 5B) the trajectory of the substrate is blocked at either side in a distance of 7–10 amino acids on either side of the position of the catalytic triad. Several of the other models also show structures blocking the substrate from fitting into the active site. The regions of the PrtP enzyme blocking at the access of the substrate are loops at either side of the catalytic triad one loop centered around residue 320 and the other around residue 429. These interactions are visualized in Figures 5B,C.

The possible interaction between substrate and the PrtP enzyme at domains further downstream has previously been suggested (Vos et al., 1991; Exterkate et al., 1993; Siezen et al., 1993; Børsting et al., 2015). From the iTAS_4 and iTAS_5 models, this region could likely be the site of interactions with residues in the region 930–950 as illustrated in Figure 5D.

### Fibronectin Like Domains, Domain A + B

**FIGURE 6:**
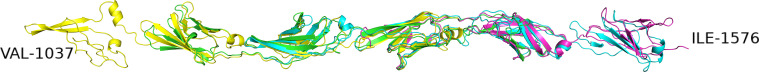
Possible fibronectin like structure of the B domain established as a superposition of four overlapping structures build by Swiss model using a monomer of 4p99 as template. Only the six last units of the nine (based on Figure 1) fibronectin like structures could be fitted to the 4p99 template and only the five last gave a complete fit to this structure. The four overlapping structures are colored in yellow, green, cyan, and magenta.

Four of the six models described in section “The proteinase domain” extends beyond the catalytic domain and covers also the A domain as defined by Siezen (1999) and the Phyre2 structure extends well into the B domain before turning into an unstructured leash. However, the regularity of the patterns in the contact maps obtained with RaptorX (cfr. Figure 1) prompted us to conduct a specific analysis of this region. As listed in Table 2; Phyre2, Swiss Model, and iTASSER all identify homology to a calcium-stabilized adhesin from the Antarctic bacterium *Marinomonas primoryensis* of which the structure has been determined and listed under the accession 4p99 in the PDB database. The reported structure is for a unit cell containing a tetramer of four antiparallel chains of identical structure. Each of the four chains contain four fibronectin like domains surrounded by calcium ions (Vance et al., 2014). When using this structure as template for molding the structure of MS22337 PrtP, Swiss Model proposes two models which are basically identical. One model corresponds to using the 4p99 structure on the last four (6–9) “aeroplane like patterns” in the contact map (cf. Figure 1). In the other model the same mold is just shifted one pattern upstream to cover “aeroplane” 5–8. In order to test if more of the patterns could be squeezed into the same mold we used a monomer chain from 4p99 as template on overlapping segments of the PrtP sequence. The resulting structure of 6 fibronectin like domains is shown in Figure 6.

**FIGURE 7:**
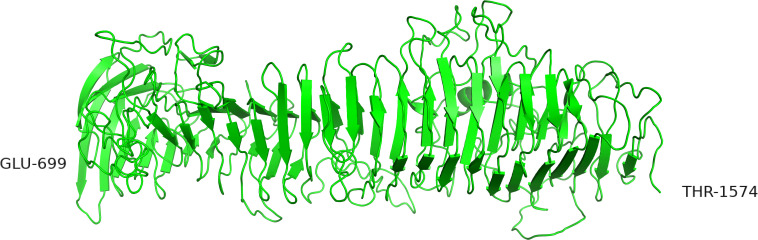
Alternative structure of the AB domain modeled by iTASSER. In this structure the β-sheets are organized in parallel orientations.

The region was also modeled by submitting the sequence from 699 to 1574 to the iTASSER server. The highest-ranking model was a structure resembling a tube build of parallel β-sheets with three faces organized as an α-helical solenoid with three β-strands per turn (Figure 7). The templates selected by i-TASSER to build this model are mainly 4rm6 and 4om9, a hemopexin binding protein from *Haemophilus influenza* and a plasmid encoded auto-transporter enterotoxin from *Escherichia coli* respectively. This tube-like structure is suggestive for a function of the A and B domains as a drain, funneling the peptides produced by the enzyme down toward the C-terminus.

The two alternative structures of Figures 6, 7 seem to be very different and mutually exclusive. However, both models seem to have biological relevant implications like maintaining a distance to the cell surface and a stability depending on the presence of calcium ions. The two models might represent different states and the function of this region might be a rod able to metamorphose into a tube transporting peptides.

### The Helical Domain, H

**FIGURE 8:**
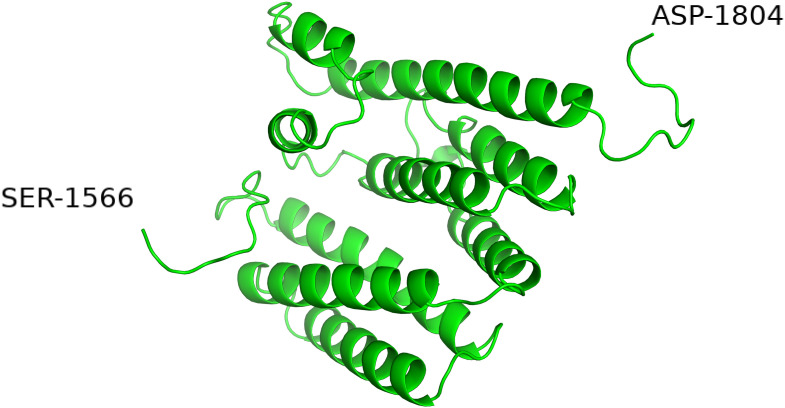
The H domain of *Lactococcus lactis* PrtP modeled by RaptorX.

The domain between residue 1570 and 1800 was termed the H-domain by Siezen due to the predicted helical secondary structure (Siezen, 1999). The contact map produced by RaptorX (Figure 1) also give an immediate impression of this region being helical in the entire length with the helixes being folded several times to give parallel and antiparallel interactions between helixes. The highest-ranking model proposed by Raptor X is shown in Figure 8.

### Domain W

The W domain is the last domain before the anchor domain. The W domain shows the largest variability of the *L. lactis* PrtP domains. The W domain varies both in sequence and in length. The length of the W domain ranges from 63 to 183 amino acids as the domain is composed of a unit of 60 amino acids repeated from 1 to 3 times with strains having 1, 1.5, 2, or 3 repeats of the basic element. Variability in sequence is seen at 11 positions within the basic unit. However, differences within repeats in the same strain is typically limited to two positions.

The function of the W domain was hypothesized to be a cell wall spanning domain allowing the proteinase to raise above the S-layer of the cell wall (Siezen, 1999). However, it has later been shown that the W-domain is involved in cell adhesion, either between *Lactococcus* cells or between lactococci and epithelia cells (Gajic, 2003). It has recently been shown that *L. lactis* expressing PrtP adheres to mucin and fibronectin (Radziwill-Bienkowska et al., 2017). It is therefore reasonable to assume that the W domain is an adhesin, and that the function of this domain during growth in milk might be to bind the casein micelle probably through binding to kappa casein located at the surface of the casein micelle.

The contact map (Figure 1) did not reveal any interactions with or within the W region, and neither of the servers listed in Table 1 gave good hits for homologous structures and they did not produce structure models with high predictive values. iTasser detected some homology to an adhesin, BoaA, from *Burkholderia pseudomallei* with structure 3s6l (Balder et al., 2010). This structure can be used as template for molding a possible structure for the MS22337 PrtP W-domain. This structure is shown in Supplementary Figure S3. However, the selection of this template is mainly based on:

• the template being an adhesin
• the structure can easily be varied in length by multiples of the turn

The structure in Supplementary Figure S3 has a pitch of 14 amino acids per turn in the solenoid. A pitch of 15 might have been more appealing as this would give a better match with repeat units of length 60. In the lack of a better structure for W, we will use the structure of Supplementary Figure S3 for the assembly of the complete structure of PrtP.

## Discussion

The purpose prompting us toward assembling a model for the structure of the cell wall proteinase of *Lactococcus lactis* was to understand the behavior of this bacterium during growth in milk. Differences in the PrtP enzyme seems to have a profound influence on the speed of acidification and to influence the ability of the bacterium to grow in different types of milk. We propose for the first time a structural model for the entire cell envelope proteinase of *Lactococcus lactis.* The model is constructed from the PrtP amino acid sequence of strain MS22337 deduced from an Illumina whole genome DNA sequence of this strain. We have used a combination of four different structure prediction servers to generate several models for overlapping segments of this large enzyme. The output from the different modeling algorithms gave structures showing differences that might represent alternative states of the enzyme. These differences have inspired us to propose a model for how the enzyme interacts with substrate during growth in milk.

### Implications for the Proteolytic System of LAB

**FIGURE 9:**
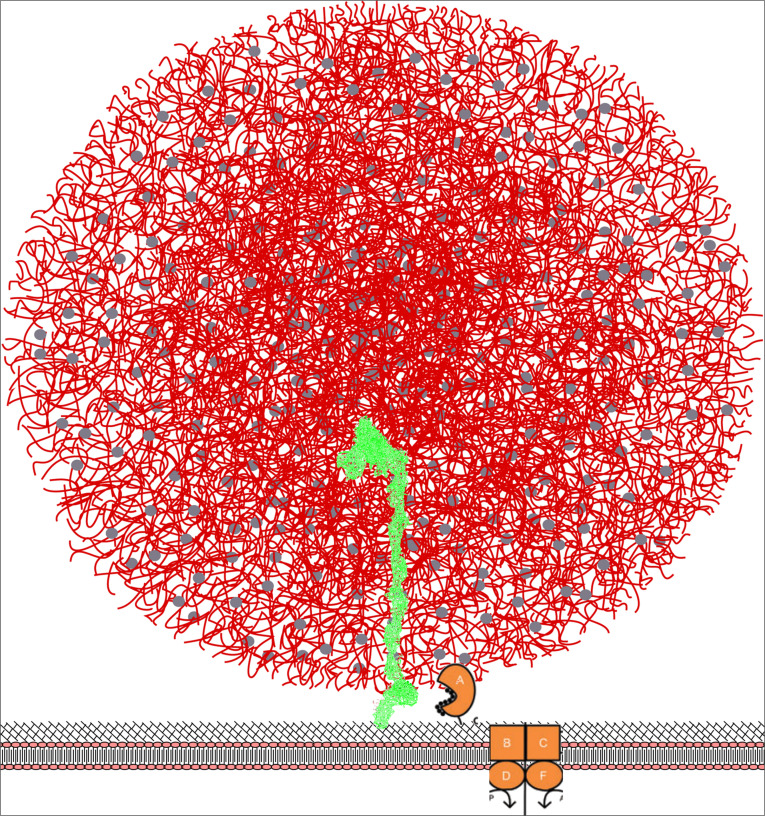
Model for the surface bound PrtP enzyme (shown in green) hydrolyzing caseins within the casein micelle. PrtP is covalently attached to the surface of the bacterium through a C-terminal threonine joined to peptidoglycan. The height of the enzyme is approximately 40 nm with the N-terminal proteinase domain at the top of molecule. The casein micelle (shown in red) has a diameter of 100 nm and is presented as the “Holt model” (Holt, 2016). We propose that the bacterium attach to the surface of the micelle and thereby position the proteinase domain in an environment of rich in casein substrates. The oligopeptide transporter proteins OppA, OppB, OppC, OppD, and OppF are shown as cartoons inspired by Doeven et al. (2005).

The model of PrtP emerging by combining the various domain structures seems to be a large protease with the proteinase domain sitting on top of a high structure being either a rod of fibronectin like units or a tube-like solenoid structure (Figure 9). The height of the enzyme will obviously depend on the shape of the stalk, with the tube structure being shorter than the fibronectin like structure, if only one fibronectin domain at the time adopts a barrel-like structure, the difference in height would be minimal. The total height of PrtP will be in the range of 200–400 Å, equal to 20–40 nM.

It is interesting to compare the dimension of the enzyme to the dimensions of the other components of milk, particularly the casein micelle, which must be the actual substrate during the early growth-phase in milk. Caseins are organized into micelles of quite variable size. A median size of 150 nM has been reported for cow milk whereas camel milk has considerable larger casein micelles (Horne, 2008; Broyard and Gaucheron, 2015). Different models for the internal structure of the casein micelle has been proposed. In spite of the differences, all models include nanoclusters of calcium phosphate kept in suspension by caseins. The surface of the micelle is covered by kappa casein having a hydrophilic glycosylated c-terminal half (Holt, 2016). In Figure 9 the PrtP and casein micelles models are superimposed in the same relative scale. The size of the bacterium will be approximately 10 times the size of a casein micelle.

It is evident that a configuration with the proteinase more or less permanently submerged into a casein micelle would give the cell several advantages. Substrate is readily available; the peptide import will be more efficient; the cell might reserve the peptides for own use rather than supporting neighboring non-proteolytic cells; the energetics of protein hydrolysis plus peptide uptake would seem to be advantageous compared to a dissociated protease.

The main peptide import system used by *Lactococcus lactis* is the oligo peptide transporter Opp able to transport peptides up to the length of 18 amino acids (Doeven et al., 2005). The uptake of a molecule by an ABC transporter will typically cost two moles of ATP per mole of substance imported (Locher, 2016; Scheepers et al., 2016). Hydrolyzing peptides inside the cell might allow the bacterium to capture the energy released by the hydrolysis, and for the longer peptides, this might result in a net gain of energy as the energy released can exceed the energy required to re-phosphorylate two moles of ATP (Martin, 1998; Bergman et al., 2010). Surplus amino acids can be used as fuel for antiporters, to import other nutrients or to export protons allowing production of ATP via the ATPase system. For LAB this route of ATP generation might be particularly favored under acidic condition as protein hydrolysis below pH 6 would “consume” protons and reduce acidity.

The proteolytic system might accordingly play a role in energy production and not only in satisfying the need for nitrogen. If the cell also captures the energy stored in the phosphoserines of the caseins, this contribution might actually be quite substantial.

The optimal proteinase for growth in milk would thus seem to be a protease able to grab entire micelles and showing a relatively broad specificity allowing hydrolysis of caseins at any part of the molecule. The resulting peptides should preferably be shorter than 18 amino acids to allow uptake. However, the peptides should be as long as possible to minimize the energy required to import the peptides. Ideally, the enzyme should be able to hold on to the peptides until they can be handed over to the peptide binding protein OppA which subsequently pass it on to the OppBC complex. In light of this, it is tempting to propose that the hydrolyzed peptides are trickling down the shaft of domain A + B either along the outside or within a “peristaltic tube.” Upon reaching the H domain, the peptides are stocked within the bundle of helices until OppA comes along to collect peptides. In this model, the logical role of the W domain would be to bind to the surface of the casein micelle, possibly via the glycosylated kappa casein.

This model for the functioning of PrtP during growth in milk could also explain the reduced acidification rate in camel milk. Larger casein micelles would allow fewer micelles to attach to the surface of the bacterium. As micelles of camel milk are four times bigger than in bovine milk, the maximal number of micelles accommodated on the surface would be reduced by approximately 16-fold.

### Matching the Model Toward Published Research

All dairy associated PrtP enzymes utilize caseins and leave whey proteins untouched. Some PrtP enzymes (P_I_ type) prefer β-casein and all types cleave β- casein at multiple sites with no obvious specificity (Visser et al., 1986; Exterkate et al., 1993; Børsting et al., 2015). Researchers at NIZO and University of Groningen have over the last three decades contributed considerably to the understanding of the specificity of the cell wall proteinases of *Lactococcus.* They have characterized the diversity in specificity of natural enzymes and they have used protein engineering to explore the molecular basis of the specificity. Mainly four types of assays have been used: (1) growth rate in reconstituted milk of strains expressing the proteinase in question, (2) hydrolysis of intact α_S__1_ casein and β- casein, (3) hydrolysis of chromogenic peptide substrates containing a *p*-nitroaniline at the C-terminal end, and (4) hydrolysis pattern of a 23 amino acid long peptide from the N-terminus of α_S__1_ casein. A body of knowledge and a large number of enzymes with altered properties have been the result of this research. However, it seems that it has been somewhat elusive to get a firm grasp on the specificity of lactocepin. Maybe we need to make a slight shift in our approach to understand the specificity of this type of proteases. Understanding protease specificity based on the Schechter and Berger model for the active site of papain (Schechter and Berger, 1967) has been very successful for a long range of proteases where the amino acids around the cleavage site determine the specificity. This model has also been guiding the research on the CEP enzymes. The underlying assumption has been that if we understand all the cleavage sites we will understand the enzyme.

Several results point toward this being a misconception:

• The CEP enzymes are rather unspecific and able to cleave at almost any position in β- casein (Visser et al., 1988, 1991, 1994; Juillard et al., 1995a; Børsting et al., 2015)
• The CEP enzymes are very selective regarding short peptide substrates. Most chromogenic substrates are not cleaved at all; and the few substrates which are cleaved, require particular salt concentrations; and the specific activity is orders of magnitude lower than for subtilisin (Exterkate, 1990)
• The CEP enzymes cleave only at very few positions within the peptide α_S__1_(1–23) (Exterkate, 1990)
• The CEP enzymes are selective regarding which caseins to hydrolyze and they leave whey proteins unhydrolyzed (Visser et al., 1986).

This seemingly contradictory behavior of an enzyme being unspecific and yet very selective can be reconciled if the enzyme is non-selective regarding the site cleaved but selective regarding the length or the nature of the substrate. An enzyme with these properties would also fit the needs of the bacterium for growth in milk.

We find that the model emerging from our investigations seems to support such a mechanism:

• The active site is similar to subtilisin which is a rather unspecific enzyme. However, in PrtP the enzyme is in the off position due to a slight displacement of the histidine of the catalytic triad
• The catalytic cleft has at either site an obstruction which might function as push-buttons needing to be activated by substrate to activate and switch on the histidine. This feature might assure that short peptides are not being “over degraded.” Long peptides might be cleaved at several positions whereas short peptides are not cleaved at all.
• Entire proteins might require auxiliary binding domains to allow for a bulky protein to squeeze into the catalytic cleft. This function might be served by the area on the surface of the fibronectin domains in the A-domain (Figure 5D)

This model fits well also with the published protein engineering investigations.

**FIGURE 10:**
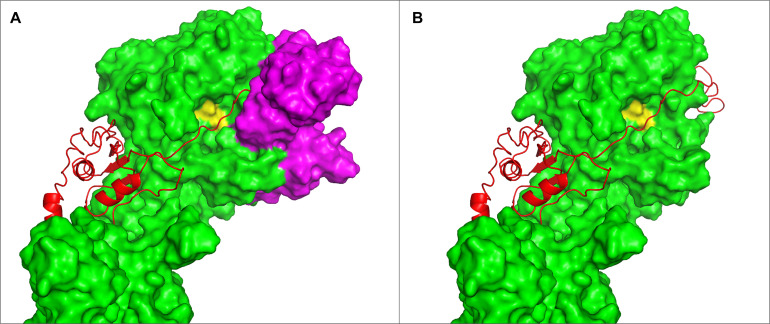
Position of the dispensable loop of Bruinenberg et al. (1994b). The structure of PrtP iTAS_4 model is shown as surface in green. Substrate positioned in the catalytic cleft is shown as red cartoon. The catalytic triad is shown as yellow on the surface of the enzyme. **(A)** The loop from 423 to 573 is shown in magenta, on **(B)** the entire loop has been removed. Residues 422 and 574 are quite close in the model (10 Å distance).

Vos et al. (1991) conducted swapping of domains between the SK11 and the Wg2 proteinases and demonstrated that a two amino acid difference in the A domain are having a profound effect on the preference for α_S__1_-casein or β- casein (Vos et al., 1991). The coordinates of the two amino acids are 747 and 748 in the mature form of the enzyme. This corresponds to 932 and 933 in the numbering on the MS22337 sequence, a position within the “blue area” on Figure 5D. The two amino acids in SK11 are arginine and lysine whereas Wg2 has leucine and threonine. The much stronger positive charge of the SK11 proteinase in this region might determine how the enzyme interacts with the negatively charged caseins and it is tempting to speculate that the clusters of phosphoserines might influence how the two enzymes make the first attack on the caseins. Vos et al. (1991) also demonstrated that differences within the N-terminal 173 amino acids also contributed to the preference for α or β- casein. Bruinenberg et al. (1994b) showed that an entire loop of the SK11 proteinase is dispensable by removal of 151 residues between position 238 and 388 (423–573 in this papers coordinates). The mutant proteinase supported growth in milk although with a reduced growth rate and the mutant retained the ability to hydrolyze α_S__1_-casein (Bruinenberg et al., 1994b). Figure 10A shows the position of this dispensable loop on our enzyme model and Figure 10B shows the appearance of the enzyme without this loop. The residues just before and just after the loop are in our model quite close, with a distance of 10 Å and the structure of the remaining molecule might therefore be only minimally disturbed. The loop is not close to the site in the A domain demonstrated to be essential for the affinity for α_S__1_ casein, and this might explain why the deletion mutant retains the P_III_ type specificity. The change might give easier access to the active site, and it would be interesting to analyze if the average length of products has changed.

In a different study Bruinenberg et al. (1994a) determined an-other shorter loop to be essential. Residues 205–219 (391–405 in our coordinates) could not be deleted without loss of enzyme activity whereas a triple mutant with substitutions of amino acids in both ends caused almost no change in activity (Bruinenberg et al., 1994a). In our model, the ends of this loop are separated by 24 Å and complete removal of the loop would most likely cause structural changes.

In a third engineering study, the same group investigated the effect of changes at specific positions of the SK11 proteinase. These positions were AKT(137–139), N166, S433, and K748 corresponding to position 322–324, 351, 618, and 933 in the MS22337 PrtP sequence. Except for the serine, which is the serine of the catalytic triad, all the positions could be mutated without losing the proteolytic activity of the enzyme. Change of the serine of the catalytic triad to an alanine led to complete inactivation of the enzyme. The AKT triplet could even be deleted or have insertions of 6 or 12 amino acids and still support growth with only minimal reduction in growth rate (Siezen et al., 1993). The AKT is located in one of the regions shown in Figure 5 to change position at contact with substrate. In our proposed model this contact with substrate could be the trigger switching the histidine to the on-position in the active site. It is interesting that the residues between 322 and 324 can be deleted and mutated without loss of activity. This might support the conclusion that this region of the molecule is adopting variable conformations. It certainly must lead to the conclusion that these residues do not play an essential role in recognizing a specific cleavage site. If the region serves a role as sensor of the length of substrate to avoid digesting small peptides even further, one would expect the phenotypes of the mutants to lead to altered length requirements for the substrate and give a different mix of positions of the actual cleavage sites. This phenotype was exactly what was observed by Siezen et al. (1993) Wild-type SK11 PrtP cleave α_S__1_(1–23) at three sites between 16 and 23 with the site closest to the N-terminus being between 16 and 17. Mutant GLA is able to cleave closer to the N-terminus at bond 13–14, mutant GDT goes as close to the N-terminus as the Wg2 PrtP and cleaves at bond 8–9, whereas mutant GPP is changed in the other direction to give a longer product by only cleaving at one site between 21 and 22 (Siezen et al., 1993).

An interesting aspect of PrtP of *Lactococcus lactis* is the need for a maturation protein PrtM for activity (Vos et al., 1989b). PrtM is a prolyl *cis/trans* isomerase needed as a chaperone for PrtP to attain activity (Siezen, 1999). It is not known which of the 69 prolines in PrtP are depending on PrtM to attain the active conformation. That the prolines of domain W should be the target, as suggested by Siezen (1999)is not likely, as truncated versions lacking W also depend on PrtM (Vos et al., 1989b). The proteolytic domain must depend on some of the critical prolines as autocatalytic processing of PrtP does not occur in absence of prtM (Vos et al., 1989b).

The templates used for modeling the structure of *Lactococcus lactis* MS22337 PrtP by iTASSER, Phyre2, and Swiss model come from the streptococcal C5a proteases ScpA and ScpB from *Streptococcus pyogenes* and *Streptococcus agalactiae* respectively (Brown et al., 2005; Kagawa et al., 2009). The structure determined by Brown et al. (2005) was obtained with a recombinant mutant protein expressed in *Escherichia coli* with the asp and ser of the catalytic triad replaced by alanines and without the pro-peptide. This structure has the histidine of the catalytic triad separated by 20 Å from the active position (Brown et al., 2005). The structure determined by Kagawa et al. (2009) was determined on a recombinant protein produced in *E. coli* containing the pro-peptide and only omitting the signal peptide. No substitutions were made and the protein had enzymatic activity (Kagawa et al., 2009). It is an open question if the different position of the histidine residue has biological relevance or if it is due to an artifact caused by the absence of the pro-peptide. The pro-peptide of subtilisin is a catalyst for correct folding of the active enzyme (Zhu et al., 1989). It is plausible that the pro-peptide serves a similar role for these much larger proteases and that this explains the extraordinary length of the pro-peptides. The C5 proteinase is a very specific proteinase with only one known substrate complement factor C5a, a 74 amino acid peptide (Kagawa and Cooney, 2013). Kagawa et al. (2009) modeled the interaction between the C5a protease and the C5a peptide and concluded that the specificity arises from complementarity between the surface of the Fn2 domain and the core structure of the peptide (Kagawa et al., 2009). Eighteen amino acids of C5a peptide between 1 and 64 could form hydrogen-, ionic-, and hydrophobic interactions with 24 amino acids on the proteinase in the region between residue 751–887 (Kagawa et al., 2009). This region would correspond to the region between residues 857–993 in MS22337 PrtP, and thus include “the blue region” of Figure 5D which also include the residues shown to determine the SK11 PrtP preference for α_S__1_ casein.

The role of domains B, H, and W in determining the specificity of CEP has been investigated by Bruinenberg et al. (2000), and they concluded that these domains are dispensable for protease activity and that they have no role in determining the specificity of the CEP enzyme. These results are important, but leaves the question of the actual function unanswered. It seems unlikely that such large domains would be maintained through evolution if they were entirely dispensable. The B domain contains the protease sensitive site exposed in absence of Ca ions and leading to autocatalytic release of the CEP from the cell wall (Bruinenberg et al., 1994a). We here propose that the function of the B domain is to position the proteinase deeply into the casein micelle. A low calcium concentration will indicate that the proteinase does not reside in a casein micelle, and under such conditions, the release of the protease might be advantageous. We suggest that the H domain might be a domain stocking the peptide products until received by OppA.

The function of domain W has been the subject of very little experimental investigation. Most researchers seem to accept the plausible suggestion of this domain being a spacer allowing the CEP enzyme to raise above the various surface layers of Gram positive bacteria (Chapot-Chartier et al., 2010; Chapot-Chartier and Kulakauskas, 2014; Mercier-Bonin and Chapot-Chartier, 2017). However, a study designed to determine the required number of W-units needed to lift an antigen above the surface layers gave the surprising result that the W-domain is an adhesin able to bind to itself and to epithelial surfaces (Gajic, 2003). In a different study the adhesive properties of PrtP were found to result in adhesion to polystyrene, mucin, and fibronectin (Radziwill-Bienkowska et al., 2017). The actual structure for the W-domain proposed by us could be far from the true structure as we picked this structure among other weak candidates based on the function as adhesin and because the structure seems to fit well with a domain which can be expanded by repeating the same unit or even fractions of the unit. The exact structure of W is not critical for our model but the function as an adhesin binding to kappa casein would be essential.

## Conclusion

From the sequence of the *Lactococcus lactis* MS22337 cell envelope proteinase, PrtP, we have modeled a structure for the complete enzyme of 1942 amino acids. Based on the model we could assign probable functions for the domains of this multi domain enzyme. These predictions are useful for guiding further experimental investigations of cell wall proteinases of lactic acid bacteria, including pathogens. The model has a general implication for serine proteases carrying a PA domain and fibronectin like domains downstream for the protease domain. This combination of domains seems to allow evolution of almost any type of specificity as substrate recognition at a fibronectin like domain activates an unspecific protease residing at a separate domain.

## Data Availability Statement

The datasets presented in this study can be found in online repositories. The names of the repository/repositories and accession number(s) can be found in the article.

## Author Contributions

EH conceived the research strategy, conducted the modeling analyses, and drafted the manuscript. PM contributed with expertise on protein modeling particularly the use of evolutionary algorithms and participated in writing the manuscript. Both authors contributed to the article and approved the submitted version.

## Conflict of Interest

The authors declare that the research was conducted in the absence of any commercial or financial relationships that could be construed as a potential conflict of interest.

## Footnotes

1 pfam.org

2 https://pymol.org

3 http://zhanglab.ccmb.med.umich.edu/TM-score

4 https://modelarchive.org/doi/10.5452/ma-qijqu

5 https://modelarchive.org/doi/10.5452/ma-jnx5p

6 https://modelarchive.org/doi/10.5452/ma-04l8v

7 https://modelarchive.org/doi/10.5452/ma-59xz0

8 https://modelarchive.org/doi/10.5452/ma-9zlh3

9 https://modelarchive.org/doi/10.5452/ma-298jo

## References

[B1] Abdelgadir W. Nielsen D. S. Hamad S. Jakobsen M. (2008). A traditional Sudanese fermented camel’s milk product, gariss, as a habitat of *Streptococcus infantarius* subsp. infantarius. *Int. J. Food Microbiol.* 127 215–219. 10.1016/j.ijfoodmicro.2008.07.008 18774196

[B2] Bachmann H. Starrenburg M. J. C. Molenaar D. Kleerebezem M. van Hylckama Vlieg J. E. T. (2012). Microbial domestication signatures of *Lactococcus lactis* can be reproduced by experimental evolution. *Genome Res.* 22 115–124. 10.1101/gr.121285.111 22080491 PMC3246198

[B3] Balder R. Lipski S. Lazarus J. J. Grose W. Wooten R. M. Hogan R. J. (2010). Identification of *Burkholderia mallei* and *Burkholderia pseudomallei* adhesins for human respiratory epithelial cells. *BMC Microbiol.* 10:250. 10.1186/1471-2180-10-250 20920184 PMC2955633

[B4] Bergman C. Kashiwaya Y. Veech R. L. (2010). The effect of pH and free Mg2+ on ATP linked enzymes and the calculation of Gibbs free energy of ATP hydrolysis. *J. Phys. Chem. B* 114 16137–16146. 10.1021/jp105723r 20866109

[B5] Berhe T. Ipsen R. Seifu E. Kurtu M. Y. Eshetu M. Hansen E. B. (2018). Comparison of the acidification activities of commercial starter cultures in camel and bovine milk. *LWT* 89 123–127. 10.1016/j.lwt.2017.10.041

[B6] Børsting M. W. Qvist K. B. Brockmann E. Vindeløv J. Pedersen T. L. Vogensen F. K. (2015). Classification of *Lactococcus lactis* cell envelope proteinase based on gene sequencing, peptides formed after hydrolysis of milk, and computer modeling. *J. Dairy Sci.* 98 68–77. 10.3168/jds.2014-8517 25465631

[B7] Bragason E. Svendsen C. A. Guya M. E. Berhe T. Hansen E. B. (2020). Draft Genome Sequences of *Lactococcus lactis* strains MS22314, MS22333, MS22336, and MS22337, Isolated from Fermented Camel Milk in Ethiopia. *Microbiol. Resour. Announc.* 9:e00862-20. 10.1128/MRA.00862-20 33214295 PMC7679088

[B8] Broadbent J. R. Steele J. L. (2013). Lactocepin: the cell envelope-associated Endopeptidase of lactococci. *Handb. Proteolytic Enzym.* 3 3188–3195. 10.1016/B978-0-12-382219-2.00703-1

[B9] Broadbent J. R. Strickland M. Weimer B. C. Johnson M. E. Steele J. L. (1998). Peptide accumulation and bitterness in cheddar cheese made using single-strain *Lactococcus lactis* starters with distinct proteinase specificities. *J. Dairy Sci.* 81 327–337. 10.3168/jds.S0022-0302(98)75581-X

[B10] Brown C. K. Gu Z.-Y. Matsuka Y. V. Purushothaman S. S. Winter L. A. Cleary P. P. (2005). Structure of the streptococcal cell wall C5a peptidase. *Proc. Natl. Acad. Sci. U.S.A.* 102 18391–18396. 10.1073/pnas.0504954102 16344483 PMC1317908

[B11] Broyard C. Gaucheron F. (2015). Modifications of structures and functions of caseins: a scientific and technological challenge. *Dairy Sci. Technol.* 95 831–862. 10.1007/s13594-015-0220-y

[B12] Bruinenberg P. G. de Vos W. M. Siezen R. J. (1994a). Prevention of C-terminal autoprocessing of *Lactococcus lactis* SK11 cell-envelope proteinase by engineering of an essential surface loop. *Biochem. J.* 302 957–963. 10.1042/bj3020957 7945226 PMC1137323

[B13] Bruinenberg P. G. Doesburg P. Alting A. C. Exterkate F. A. Vos W. M. D. Siezen R. J. (1994b). Evidence for a large dispensable segment in the subtilisin-like catalytic domain of the *Lactococcus lactis* cell-envelope proteinas. *Protein Eng. Des. Sel.* 7 991–996. 10.1093/protein/7.8.991 7528919

[B14] Bruinenberg P. G. De Vos W. M. Siezen R. J. (2000). Deletion of various carboxy-terminal domains of *Lactococcus lactis* SK11 proteinase: effects on activity, specificity, and stability of the truncated enzyme. *Appl. Environ. Microbiol.* 66 2859–2865. 10.1128/AEM.66.7.2859-2865.2000 10877779 PMC92084

[B15] Chapot-Chartier M. P. Kulakauskas S. (2014). Cell wall structure and function in lactic acid bacteria. *Microb. Cell Fact.* 13:S9. 10.1186/1475-2859-13-S1-S9 25186919 PMC4155827

[B16] Chapot-Chartier M. P. Vinogradov E. Sadovskaya I. Andre G. Mistou M. Y. Trieu-Cuot P. (2010). Cell surface of *Lactococcus lactis* is covered by a protective polysaccharide pellicle. *J. Biol. Chem.* 285 10464–10471. 10.1074/jbc.M109.082958 20106971 PMC2856253

[B17] Cranford-Smith T. Huber D. (2018). The way is the goal: how SecA transports proteins across the cytoplasmic membrane in bacteria. *FEMS Microbiol. Lett.* 365 1–16. 10.1093/femsle/fny093 29790985 PMC5963308

[B18] de Vos W. M. Vos P. de Haard H. Boerrigter I. (1989). Cloning and expression of the *Lactococcus lactis* subsp. cremoris SK11 gene encoding an extracellular serine proteinase. *Gene* 85 169–176. 10.1016/0378-1119(89)90477-0 2515994

[B19] Doeven M. K. Kok J. Poolman B. (2005). Specificity and selectivity determinants of peptide transport in *Lactococcus lactis* and other microorganisms. *Mol. Microbiol.* 57 640–649. 10.1111/j.1365-2958.2005.04698.x 16045610

[B20] Domingo Meza-Aguilar J. Fromme P. Torres-Larios A. Mendoza-Hernández G. Hernandez-Chiñas U. Arreguin-Espinosa De Los Monteros R. A. (2014). X-ray crystal structure of the passenger domain of plasmid encoded toxin(Pet), an autotransporter enterotoxin from enteroaggregative *Escherichia coli* (EAEC). *Biochem. Biophys. Res. Commun.* 445 439–444. 10.1016/j.bbrc.2014.02.016 24530907 PMC4005925

[B21] Drici H. Gilbert C. Kihal M. Atlan D. (2010). Atypical citrate-fermenting *Lactococcus lactis* strains isolated from dromedary’s milk. *J. Appl. Microbiol.* 108 647–657. 10.1111/j.1365-2672.2009.04459.x 19663815

[B22] Drici H. (2008). Analyse Physiologique, Génétique et Moléculaire de Lactocoques Protéolytiques Issus du lait cru de Chamelle d ’ Algérie. Thesis, Université Claude Bernard Lyon, France.

[B23] Exterkate F. A. (1990). Differences in short peptide-substrate cleavage by two cell-envelope-located serine proteinases of *Lactococcus lactis* subsp. cremoris are related to secondary binding specificity. *Appl. Microbiol. Biotechnol.* 33 401–406. 10.1007/BF00176654 1366743

[B24] Exterkate F. A. Alting A. C. (1999). Role of calcium in activity and stability of the *Lactococcus lactis* cell envelope proteinase. *Appl. Environ. Microbiol.* 65 1390–1396. 10.1128/AEM.65.4.1390-1396.1999 10103227 PMC91197

[B25] Exterkate F. A. Alting A. C. Bruinenberg P. G. (1993). Diversity of cell envelope proteinase specificity among strains of *Lactococcus lactis* and its relationship to charge characteristics of the substrate-binding region. *Appl. Environ. Microbiol.* 59 3640–3647. 10.1128/aem.59.11.3640-3647.1993 8285671 PMC182510

[B26] Fugl A. Berhe T. Kiran A. Hussain S. Laursen M. F. Bahl M. I. (2017). Characterisation of lactic acid bacteria in spontaneously fermented camel milk and selection of strains for fermentation of camel milk. *Int. Dairy J.* 73 19–24. 10.1016/j.idairyj.2017.04.007

[B27] Gabed N. Yang M. Bey M. Hamed B. Drici H. Gross R. (2015). Draft genome sequence of the moderately heat-tolerant *Lactococcus lactis* subsp. lactis bv. diacetylactis Strain GL2 from Algerian. *Genome Announc* 3 13–14. 10.1128/genomeA.01334-15 26586883 PMC4653785

[B28] Gajic O. (2003). *Relationships Between MDR Proteins, Bacteriocin Production and Proteolysis in Lactococcus lactis.* PhD Thesis, University of. Groningen, Netherlands.

[B29] Gänzle M. G. (2015). Lactic metabolism revisited: metabolism of lactic acid bacteria in food fermentations and food spoilage. *Curr. Opin. Food Sci.* 2 106–117. 10.1016/j.cofs.2015.03.001

[B30] Griessl M. H. Schmid B. Kassler K. Braunsmann C. Ritter R. Barlag B. (2013). Structural insight into the giant Ca2+-binding adhesin siie: implications for the adhesion of *Salmonella enterica* to polarized epithelial cells. *Structure* 21 741–752. 10.1016/j.str.2013.02.020 23562396

[B31] Haandrikman A. J. Kok J. Laan H. Soemitro S. Ledeboer A. M. Konings W. N. (1989). Identification of a gene required for maturation of an extracellular lactococcal serine proteinase. *Environ. Microbiol.* 171 2789–2794. 10.1128/jb.171.5.2789-2794.1989 2708318 PMC209965

[B32] Holt C. (2016). Casein and casein micelle structures, functions and diversity in 20 species. *Int. Dairy J.* 60 2–13. 10.1016/j.idairyj.2016.01.004

[B33] Horne D. S. (2008). *Casein Micelle Structure and Stability*, 2nd Edn Amsterdam: Elsevier Inc, 10.1016/B978-0-12-374039-7.00005-2

[B34] Jensen P. R. Hammer K. (1993). Minimal requirements for exponential growth of *Lactococcus lactis*. *Appl. Environ. Microbiol.* 59 4363–4366. 10.1093/emboj/16.12.3533 16349136 PMC195913

[B35] Jobichen C. Tan Y. C. Prabhakar M. T. Nayak D. Biswas D. Pannu N. S. (2018). Structure of ScpC, a virulence protease from *Streptococcus pyogenes*, reveals the functional domains and maturation mechanism. *Biochem. J.* 475 2847–2860. 10.1042/BCJ20180145 30049896

[B36] Juillard V. Laan H. Kunji E. R. S. Jeronimus-Stratingh C. M. Bruins A. P. Konings W. N. (1995a). The extracellular P(I)-type proteinase of *Lactococcus lactis* hydrolyzes β-casein into more than one hundred different oligopeptides. *J. Bacteriol.* 177 3472–3478. 10.1128/jb.177.12.3472-3478.1995 7768856 PMC177051

[B37] Juillard V. le Bars D. Kunji E. R. S. Konings W. N. Gripon J.-C. Richard J. (1995b). Oligopeptides are the main source of nitrogen for *Lactococcus lactis* during growth in milk. *Appl. Environ. Microbiol.* 61 3024–3030. 10.1128/aem.61.8.3024-3030.1995 7487034 PMC167578

[B38] Kagawa T. F. Cooney J. C. (2013). “C5a Peptidase,” in *Handbook of Proteolytic Enzymes*, eds Rawlings N. D. Salvesen G. (Amsterdam: Elsevier), 3202–3208. 10.1016/B978-0-12-382219-2.00705-5

[B39] Kagawa T. F. O’Connell M. R. Mouat P. Paoli M. O’Toole P. W. Cooney J. C. (2009). Model for Substrate Interactions in C5a Peptidase from *Streptococcus pyogenes*: a 1.9 Å Crystal Structure of the Active Form of ScpA. *J. Mol. Biol.* 386 754–772. 10.1016/j.jmb.2008.12.074 19152799

[B40] Kelley L. A. Mezulis S. Yates C. M. Wass M. N. Sternberg M. J. E. (2015). The Phyre2 web portal for protein modeling, prediction and analysis. *Nat. Protoc.* 10 845–858. 10.1038/nprot.2015.053 25950237 PMC5298202

[B41] Kim J.-S. Kluskens L. D. de Vos W. M. Huber R. van der Oost J. (2004). Crystal structure of fervidolysin from fervidobacterium pennivorans, a keratinolytic enzyme related to subtilisin. *J. Mol. Biol.* 335 787–797. 10.1016/j.jmb.2003.11.006 14687574

[B42] Kliche T. Li B. Bockelmann W. Habermann D. Klempt M. de Vrese M. (2017). Screening for proteolytically active lactic acid bacteria and bioactivity of peptide hydrolysates obtained with selected strains. *Appl. Microbiol. Biotechnol.* 101 7621–7633. 10.1007/s00253-017-8369-3 28695230

[B43] Kok J. Leenhouts K. J. Haandrikman A. J. Ledeboer A. M. Venema G. (1988). Nucleotide sequence of the cell wall proteinase gene of *Streptococcus cremoris* Wg2. *Appl. Environ. Microbiol.* 54 231–238. 10.1128/AEM.54.1.231-238.1988 3278687 PMC202426

[B44] Locher K. P. (2016). Mechanistic diversity in ATP-binding cassette (ABC) transporters. *Nat. Struct. Mol. Biol.* 23 487–493. 10.1038/nsmb.3216 27273632

[B45] Makarova K. Slesarev A. Wolf Y. Sorokin A. Mirkin B. Koonin E. (2006). Comparative genomics of the lactic acid bacteria. *Proc. Natl. Acad. Sci. U.S.A* 103 15611–15616. 10.1073/pnas.0607117103 17030793 PMC1622870

[B46] Marks D. S. Hopf T. A. Sander C. (2012). Protein structure prediction from sequence variation. *Nat. Biotechnol.* 30 1072–1080. 10.1038/nbt.2419 23138306 PMC4319528

[B47] Martin R. B. (1998). Free energies and equilibria of peptide bond hydrolysis and formation. *Biopolymers* 45 351–353. 10.1002/(SICI)1097-0282(19980415)45:5<351::AID-BIP3<3.0.CO;2-K

[B48] Mercier-Bonin M. Chapot-Chartier M.-P. (2017). Surface proteins of *Lactococcus lactis*: bacterial resources for muco-adhesion in the gastrointestinal tract. *Front. Microbiol.* 8:2247. 10.3389/fmicb.2017.02247 29218032 PMC5703838

[B49] Murayama K. Kato-Murayama M. Hosaka T. Sotokawauchi A. Yokoyama S. Arima K. (2012). Crystal structure of cucumisin, a subtilisin-like endoprotease from *Cucumis melo* L. *J. Mol. Biol.* 423 386–396. 10.1016/j.jmb.2012.07.013 22841692

[B50] Radziwill-Bienkowska J. M. Robert V. Drabot K. Chain F. Cherbuy C. Langella P. (2017). Contribution of plasmid-encoded peptidase S8 (PrtP) to adhesion and transit in the gut of *Lactococcus lactis* IBB477 strain. *Appl. Microbiol. Biotechnol.* 101 5709–5721. 10.1007/s00253-017-8334-1 28540425 PMC5501904

[B51] Savijoki K. Ingmer H. Varmanen P. (2006). Proteolytic systems of lactic acid bacteria. *Appl. Microbiol. Biotechnol.* 71 394–406. 10.1007/s00253-006-0427-1 16628446

[B52] Schechter I. Berger A. (1967). On the size of the active site in proteases. I. Papain. *Biochem. Biophys. Res. Commun.* 27 157–162. 10.1016/s0006-291x(67)80055-x 6035483

[B53] Scheepers G. H. Lycklama A. Nijeholt J. A. Poolman B. (2016). An updated structural classification of substrate-binding proteins. *FEBS Lett.* 590 4393–4401. 10.1002/1873-3468.12445 27714801

[B54] Siezen R. J. (1999). Multi-domain, cell-envelope proteinases of lactic acid bacteria. *Antonie van Leeuwenhoek* 76 139–155. 10.1023/A:1002036906922 10532376

[B55] Siezen R. J. Bruinenberg P. G. Vos P. van Alen-Boerrigter I. Nijhuis M. Alting A. C. (1993). Engineering of the substrate-binding region of the sublilisin-like, cell-envelop proteinase of *Lactococcus lactis*. *Protein Eng. Des. Sel.* 6 927–937. 10.1093/protein/6.8.927 8309942

[B56] Siezen R. J. Leunissen J. A. M. (2010). Subtilases: the superfamily of subtilisin-like serine proteases. *Protein Sci.* 6 501–523. 10.1002/pro.5560060301 9070434 PMC2143677

[B57] Siezen R. J. Renckens B. Van Swam I. Peters S. Van Kranenburg R. Kleerebezem M. (2005). Complete sequences of four plasmids of *Lactococcus lactis* subsp. *cremoris SK*11 reveal extensive adaptation to the dairy environment. *Appl. Environ. Microbiol.* 71 8371–8382. 10.1128/AEM.71.12.8371-8382.2005 16332824 PMC1317451

[B58] Sodini I. Latrille E. Corrieu G. (2000). Identification of interacting mixed cultures of lactic acid bacteria by their exclusion from a model predicting the acidifying activity of non-interacting mixed cultures. *Appl. Microbiol. Biotechnol.* 54 715–718. 10.1007/s002530000460 11131401

[B59] Vance T. D. R. Olijve L. L. C. Campbell R. L. Voets I. K. Davies P. L. Guo S. (2014). Ca2+-stabilized adhesin helps an Antarctic bacterium reach out and bind ice. *Biosci. Rep.* 34 357–368. 10.1042/BSR20140083 24892750 PMC4083281

[B60] Vermeulen N. Pavlovic M. Ehrmann M. A. Ganzle M. G. Vogel R. F. (2005). Functional Characterization of the proteolytic system of *Lactobacillus sanfranciscensis* DSM 20451T during Growth in Sourdough. *Appl. Environ. Microbiol.* 71 6260–6266. 10.1128/AEM.71.10.6260-6266.2005 16204547 PMC1266010

[B61] Visser S. Exterkate F. A. Slangen C. J. de Veer G. J. C. M. (1986). Comparative study of action of cell wall proteinases from various strains of *Streptococcus cremoris* on Bovine α s1-, β-, and κ-Casein. *Appl. Environ. Microbiol.* 52 1162–1166. 10.1128/AEM.52.5.1162-1166.1986 16347215 PMC239191

[B62] Visser S. Robben A. J. P. M. Slangen C. J. (1991). Specificity of a cell-envelope-located proteinase (Pro-type) from *Lactococcus lactis* subsp, cremoris AM1 in its action on bovine//-casein. *Appl. Microbiol. Biotechnol.* 35 477–483. 1367552 10.1007/BF00169753

[B63] Visser S. Slangen C. J. Exterkate F. A. de Veer G. J. C. M. (1988). Action of a cell wall proteinase (PI) from *Streptococcus cremoris* HP on bovine β-casein. *Appl. Microbiol. Biotechnol.* 29 61–66. 10.1007/BF00258352

[B64] Visser S. Slangen C. J. Robben A. J. P. M. van Dongen W. D. Heerma W. Haverkamp J. (1994). Action of a cell-envelope proteinase (CEPIII-type) from *Lactococcus lactis* subsp. *cremoris AM*1 on bovine κ-casein. *Appl. Microbiol. Biotechnol.* 41 644–651. 10.1007/BF00167279 7765163

[B65] Vos P. Boerrigter I. J. Buist G. Haandrikman A. J. Nijhuis M. de Reuver M. B. (1991). Engineering of the *Lactococcus lactis* serine proteinase by construction of hybrid enzymes. *Protein Eng. Des. Sel.* 4 479–484. 10.1093/protein/4.4.479 1881875

[B66] Vos P. Simons G. Siezen R. J. de Vos W. M. (1989a). Primary structure and organization of the gene for a procaryotic, cell envelope-located serine proteinase. *J. Biol. Chem.* 264 13579–13585. 2760036

[B67] Vos P. van Asseldonk M. van Jeveren F. Siezen R. Simons G. de Vos W. M. (1989b). A maturation protein is essential for production of active forms of *Lactococcus lactis* SK11 serine proteinase located in or secreted from the cell envelope. *J. Bacteriol.* 171 2795–2802. 10.1128/JB.171.5.2795-2802.1989 2496115 PMC209966

[B68] Wang S. Sun S. Li Z. Zhang R. Xu J. (2017). Accurate de novo prediction of protein contact map by ultra-deep learning model. *PLoS Comput. Biol.* 13:e1005324. 10.1371/journal.pcbi.1005324 28056090 PMC5249242

[B69] Waterhouse A. Bertoni M. Bienert S. Studer G. Tauriello G. Gumienny R. (2018). SWISS-MODEL: homology modelling of protein structures and complexes. *Nucleic Acids Res.* 46 W296–W303. 10.1093/nar/gky427 29788355 PMC6030848

[B70] Xu J. (2019). Distance-based protein folding powered by deep learning. *Proc. Natl. Acad. Sci. U.S.A.* 116 16856–16865. 10.1073/pnas.1821309116 31399549 PMC6708335

[B71] Yang J. Yan R. Roy A. Xu D. Poisson J. Zhang Y. (2015). The I-TASSER Suite: protein structure and function prediction. *Nat. Methods* 12 7–8. 10.1038/nmeth.3213 25549265 PMC4428668

[B72] Zambolin S. Clantin B. Chami M. Hoos S. Haouz A. Villeret V. (2016). Structural basis for haem piracy from host haemopexin by *Haemophilus influenzae*. *Nat. Commun.* 7:11590. 10.1038/ncomms11590 27188378 PMC4873976

[B73] Zhang Y. Skolnick J. (2004). Scoring function for automated assessment of protein structure template quality. *Proteins Struct. Funct. Bioinforma* 57 702–710. 10.1002/prot.20264 15476259

[B74] Zhou X. Hu J. Zhang C. Zhang G. Zhang Y. (2019). Assembling multidomain protein structures through analogous global structural alignments. *Proc. Natl. Acad. Sci. U.S.A.* 116 15930–15938. 10.1073/pnas.1905068116 31341084 PMC6689945

[B75] Zhu X. Ohta Y. Jordan F. Inouye M. (1989). Pro-sequence of subtilisin can guide the refolding of denatured subtilisin in an intermolecular process. *Nature* 339 483–484. 10.1038/339483a0 2657436

