# Supplementary figures and images for: Modeled Structure of the Cell Envelope Proteinase of Lactococcus lactis
Source: Front Bioeng Biotechnol. 2020 Dec 22;8:613986. doi: 10.3389/fbioe.2020.613986 (PMC7783315; doi:10.3389/fbioe.2020.613986)

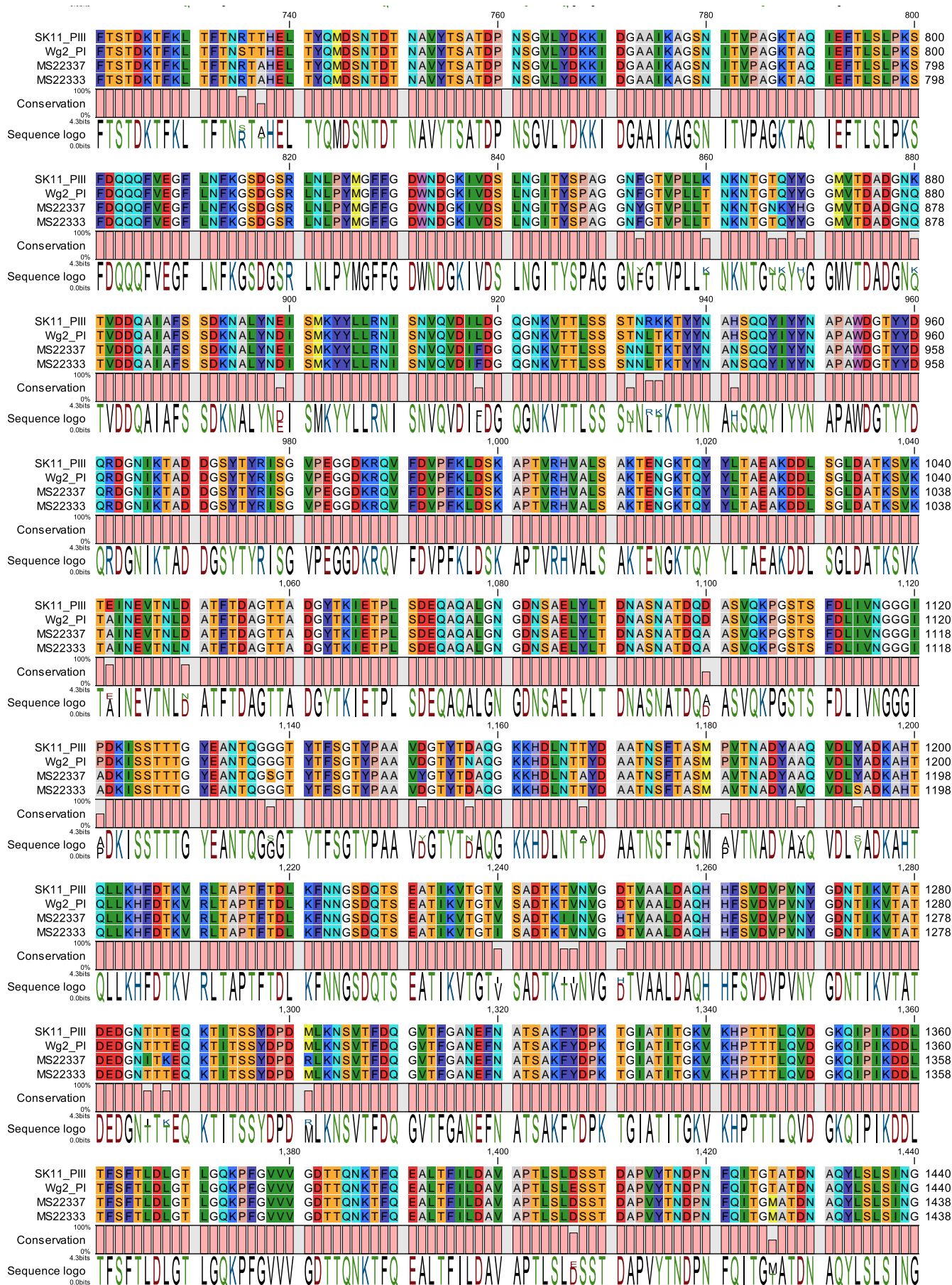

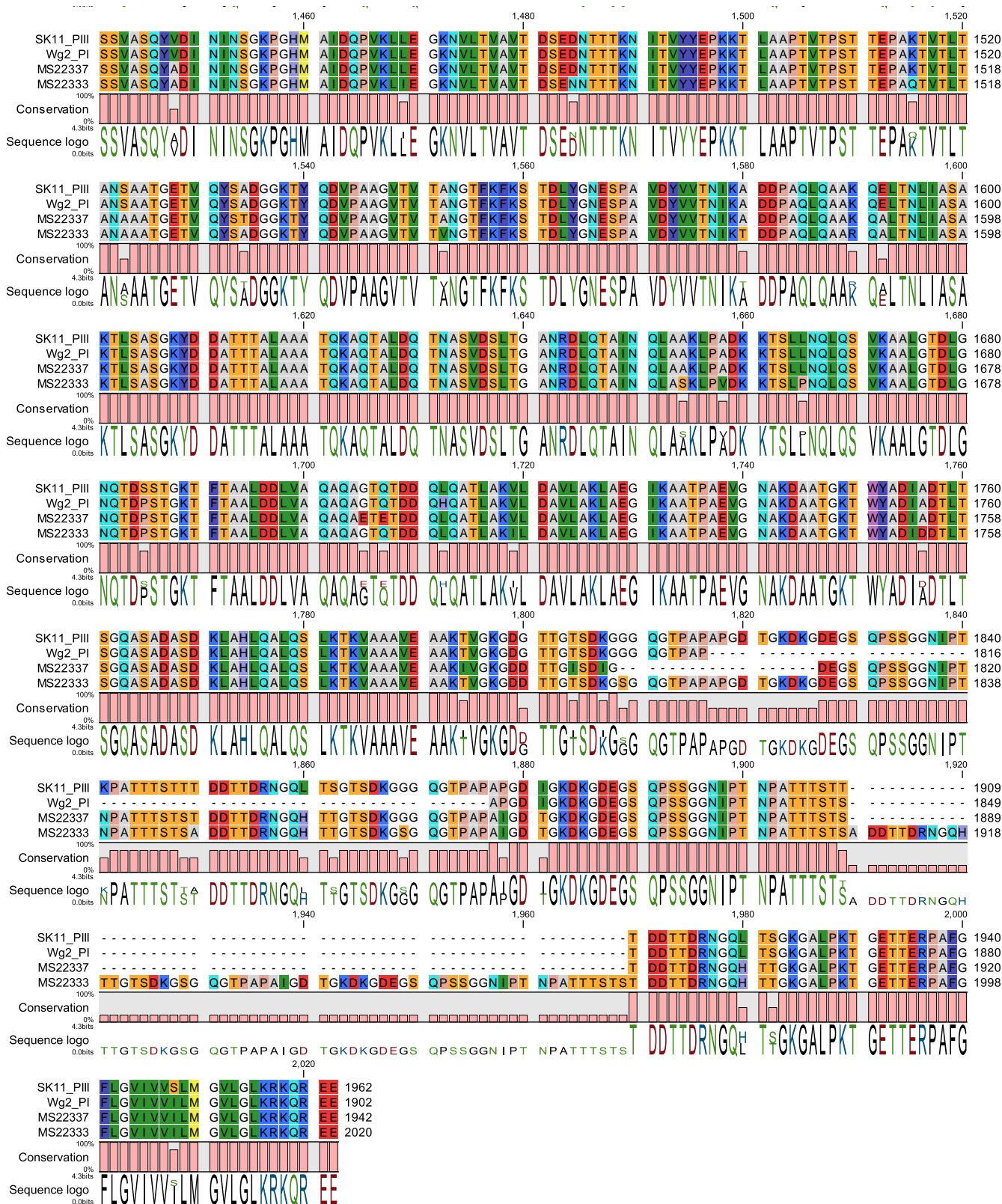

Supplement: Supplementary file 1 [file Data_Sheet_1.PDF]
