# Supplementary material for: Modeled Structure of the Cell Envelope Proteinase of Lactococcus lactis
Source: Front Bioeng Biotechnol. 2020 Dec 22;8:613986. doi: 10.3389/fbioe.2020.613986 (PMC7783315; doi:10.3389/fbioe.2020.613986)
Supplement: Supplementary file 2 [file Data_Sheet_2.PDF]

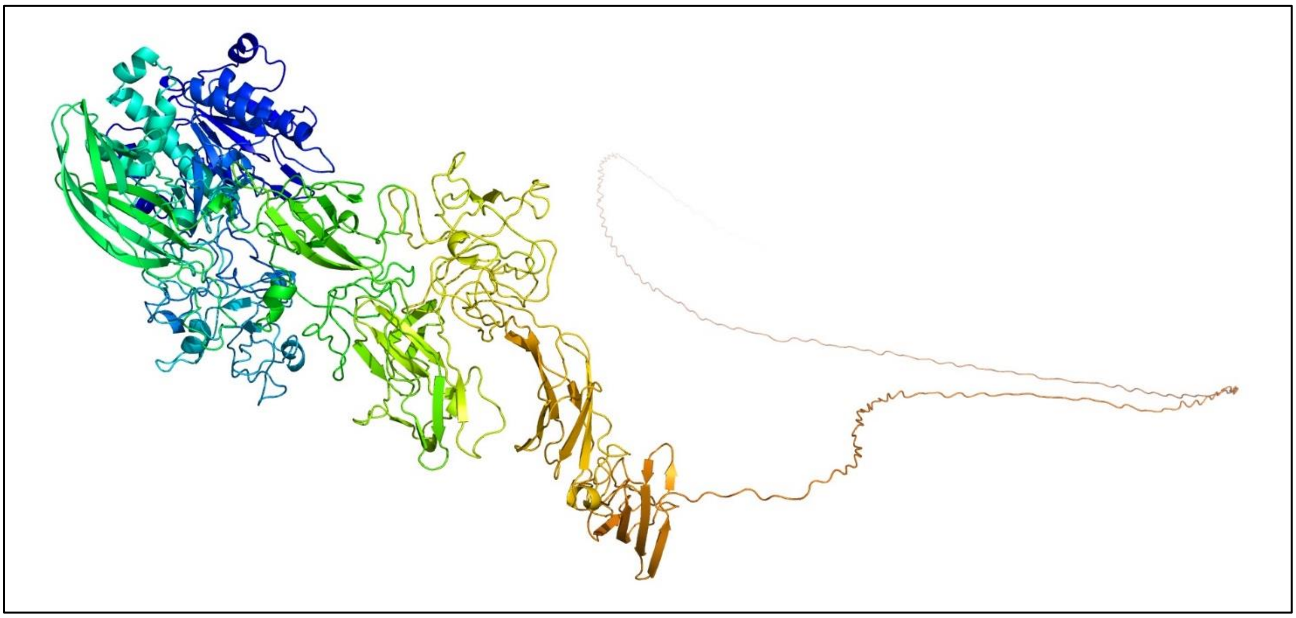

Supplementary Figure S2

Structure of the cell envelope proteinase of *Lactococcus lactis* strain MS22337 as modelled by Phyre2 based on the amino acid sequence of the mature protein deduced from the sequence of the *prtP* gene.
