# Supplementary material for: Modeled Structure of the Cell Envelope Proteinase of Lactococcus lactis
Source: Front Bioeng Biotechnol. 2020 Dec 22;8:613986. doi: 10.3389/fbioe.2020.613986 (PMC7783315; doi:10.3389/fbioe.2020.613986)
Supplement: Supplementary file 3 [file Data_Sheet_3.PDF]

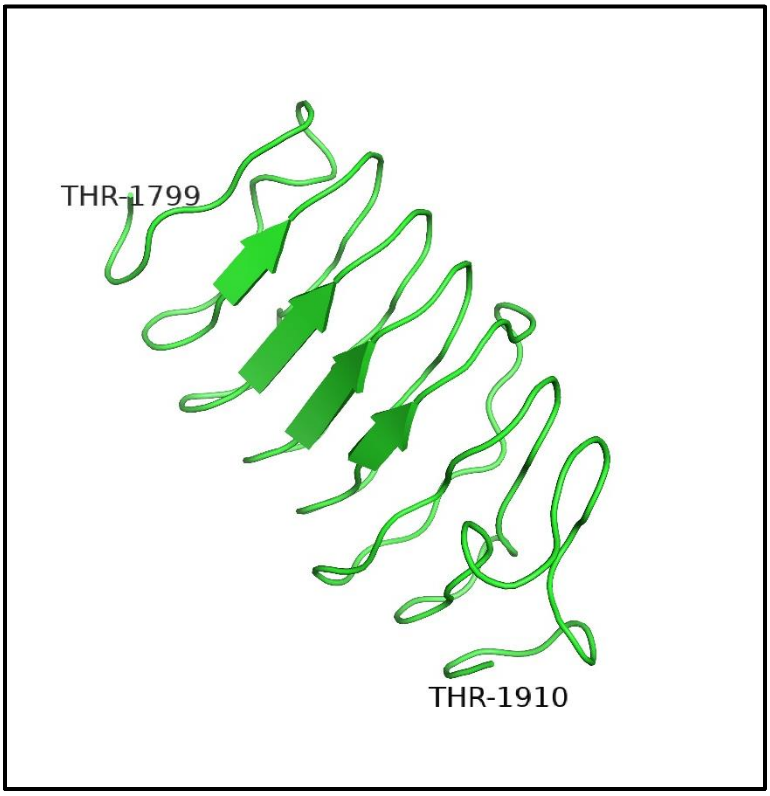

Figure S3

One of the structures proposed by iTASSER for the W domain of PrtP. None of the predicted structures had high scores and the actual structure of W could be quite different.
